# Use of Artificial Intelligence in the Identification and Diagnosis of Frailty Syndrome in Older Adults: Scoping Review

**DOI:** 10.2196/47346

**Published:** 2023-10-20

**Authors:** Daniel Velazquez-Diaz, Juan E Arco, Andres Ortiz, Verónica Pérez-Cabezas, David Lucena-Anton, Jose A Moral-Munoz, Alejandro Galán-Mercant

**Affiliations:** 1 ExPhy Research Group, Department of Physical Education Faculty of Education Sciences University of Cadiz Cádiz Spain; 2 Advent Health Research Institute Neuroscience Institute Orlando, FL United States; 3 Department of Communications Engineering University of Malaga Málaga Spain; 4 Andalusian Research Institute in Data Science and Computational Intelligence Granada Spain; 5 Department of Signal Theory, Networking and Communications University of Granada Granada Spain; 6 MOVE-IT Research Group, Department of Nursing and Physiotherapy Faculty of Health Sciences University of Cádiz Cádiz Spain; 7 Biomedical Research and Innovation Institute of Cádiz Cádiz Spain; 8 Department of Nursing and Physiotherapy Faculty of Nursing and Physiotherapy University of Cadiz Cádiz Spain

**Keywords:** frail older adult, identification, diagnosis, artificial intelligence, review, frailty, older adults, aging, biological variability, detection, accuracy, sensitivity, screening, tool

## Abstract

**Background:**

Frailty syndrome (FS) is one of the most common noncommunicable diseases, which is associated with lower physical and mental capacities in older adults. FS diagnosis is mostly focused on biological variables; however, it is likely that this diagnosis could fail owing to the high biological variability in this syndrome. Therefore, artificial intelligence (AI) could be a potential strategy to identify and diagnose this complex and multifactorial geriatric syndrome.

**Objective:**

The objective of this scoping review was to analyze the existing scientific evidence on the use of AI for the identification and diagnosis of FS in older adults, as well as to identify which model provides enhanced accuracy, sensitivity, specificity, and area under the curve (AUC).

**Methods:**

A search was conducted using PRISMA-ScR (Preferred Reporting Items for Systematic Reviews and Meta-Analyses extension for Scoping Reviews) guidelines on various databases: PubMed, Web of Science, Scopus, and Google Scholar. The search strategy followed Population/Problem, Intervention, Comparison, and Outcome (PICO) criteria with the population being older adults; intervention being AI; comparison being compared or not to other diagnostic methods; and outcome being FS with reported sensitivity, specificity, accuracy, or AUC values. The results were synthesized through information extraction and are presented in tables.

**Results:**

We identified 26 studies that met the inclusion criteria, 6 of which had a data set over 2000 and 3 with data sets below 100. Machine learning was the most widely used type of AI, employed in 18 studies. Moreover, of the 26 included studies, 9 used clinical data, with clinical histories being the most frequently used data type in this category. The remaining 17 studies used nonclinical data, most frequently involving activity monitoring using an inertial sensor in clinical and nonclinical contexts. Regarding the performance of each AI model, 10 studies achieved a value of precision, sensitivity, specificity, or AUC ≥90.

**Conclusions:**

The findings of this scoping review clarify the overall status of recent studies using AI to identify and diagnose FS. Moreover, the findings show that the combined use of AI using clinical data along with nonclinical information such as the kinematics of inertial sensors that monitor activities in a nonclinical context could be an appropriate tool for the identification and diagnosis of FS. Nevertheless, some possible limitations of the evidence included in the review could be small sample sizes, heterogeneity of study designs, and lack of standardization in the AI models and diagnostic criteria used across studies. Future research is needed to validate AI systems with diverse data sources for diagnosing FS. AI should be used as a decision support tool for identifying FS, with data quality and privacy addressed, and the tool should be regularly monitored for performance after being integrated in clinical practice.

## Introduction

Owing to significant progress in medicine and science, life expectancy has generally increased among the global population [1] and, consequently, among the older adult population [2]. Aging is a multifactorial and multiorganic process characterized by a decline in physical integrity and quality of life, along with an increased incidence of health-related issues and noncommunicable diseases [3]. Frailty syndrome (FS) is one of the most common noncommunicable diseases and is indeed one of the main causes of dependency, associated with a lower intrinsic capacity in older adults [4]. FS is associated with age, characterized by a decrease in an individual’s biological reserve and resistance to stress due to the decline in multiple systems that increases vulnerability of the individual and risk of adverse health outcomes, including disability, falls, cognitive decline, hospitalization, permanent institutionalization, and death [3]. The impact of frailty on the population is high, reaching an average of 10% for those over 65 years of age, and is defined as “the composite of all the physical and mental capacities of an individual” [5]. For this reason, a decreased intrinsic capacity produces alterations in the perception of an individual’s quality of life, a lower capacity to carry out basic daily life activities, and a loss of independence [6]. Therefore, a decline in intrinsic capacity is unmistakably one of the principal causes of frailty [7].

Currently, the most standardized and accepted definitions for the diagnosis of FS is that proposed by Fried et al [8], who established a phenotype of frailty based on weight loss, low grip strength, exhaustion, slowness, and low activity. However, they worked with biological variables, excluding other types of variables. Scientific evidence has proven that there are other factors that may lead to a process of vulnerability and frailty in older adults, such as a sedentary lifestyle, unhealthy diet, social environment, cognitive state, or existing comorbidities [9]. Nonetheless, it is agreed that frailty is a condition preceding disability and that strategies must be set for an early classification and identification of older adults into nonfrail, prefrail, and frail individuals [10]. Furthermore, scientific evidence [11] shows that an FS diagnosis is principally focused on analyzing the loss of functional capacity in older adults, usually including variables related to the musculoskeletal system, and particularly lower limb–related and lower limb–centered variables [11]. Therefore, there is evidence in relation to kinematics digital biomarkers [12]. Using these sensors, differences in the outcomes of monitored tests between frail and nonfrail individuals have been found [13], demonstrating enhanced diagnostic sensitivity and specificity compared to conventional tools such as the Fried index or the Frailty Scale [14].

Because the process for FS diagnosis is mostly focused on biological variables, the classification of older adults is extremely likely to fail due to high variability in the biological indicators [15]. For this reason, and given the multifactorial nature of FS, the use of artificial intelligence (AI) and data science is being considered when identifying and diagnosing this complex geriatric syndrome. AI may set a relationship among different variables, including biological, cognitive, kinematic, and social support, resulting in a more precise classification and addressing the complex diagnosis of a multifactorial syndrome. AI provides a set of analysis methods that, through statistics-related and automated learning techniques, enable the identification of patterns within a data set and connecting them to a specific condition [16]. Moreover, AI-based analysis techniques combine multimodal and multifactorial information, clinical data (medical imaging, questionnaires, or other data from the medical history), and nonclinical data (kinematic or physical activity monitoring data).

In the framework of AI methods, we might distinguish between those based on statistical learning (usually referred to as machine learning [17]) and those based on neural networks. In recent years, neural network–based methods have become increasingly popular owing to their significant capacity for pattern learning and standardization, particularly for certain problems, compared to conventional statistical models. Deep learning is based on the connectionist model by which the functioning of a human brain may be explained from a computational perspective. Artificial neurons are processing units designed on the bases of biological neurons that can carry out a very simple operation. Although a single neuron cannot solve any complex problem, the connectionist theory states that combining numerous neurons structured in layers—as is the case in animals’ nervous systems and particularly in the brain—results in a machine able to process data in a distributed form that can simultaneously solve hugely complex problems [18]. Currently, the most outstanding progress in AI has been made through the development of deep learning neural architectures. Within biomedicine, AI, and more particularly the deep learning–based methods, have led to significantly enhanced accuracy of image processing and classification systems, allowing for more accurate and earlier diagnoses. In fact, the potential of AI could help solve the problem of diagnosing FS, as the significant variability of FS as a multifactorial syndrome and dependent on multiple factors must be taken into account [9]. Using AI that considers a large quantity of different data, which are only managed using computational systems, will allow for a more accurate diagnosis [18]. This is more compelling when designing smart systems that are not only accurate but also provide additional information on the variables used during the classification process.

To the best of our knowledge, no scoping review exists on the role of AI in the identification and diagnosis of FS in older adults. Therefore, the primary aim of this scoping review was to analyze the existing scientific evidence on the use of AI for the identification and diagnosis of FS in older adults, as well as to identify which model provides enhanced accuracy, sensitivity, specificity, and area under the curve (AUC).

## Methods

### Design

This scoping review was conducted using the recommended PRISMA-ScR (Preferred Reporting Items for Systematic Reviews and Meta-Analyses extension for Scoping Reviews) guidelines published in 2018 [19].

### Information Sources

This scoping review was conducted between February and April 2022. The databases examined included PubMed, Web of Science (WoS), Scopus, and Google Scholar. All papers available on these databases were exported to Mendeley software, which eliminated duplicate papers. Upon completion of this first step, the screening of evidence was initiated.

### Search Strategy

The search strategy was conducted following the Population/Problem, Intervention, Comparison, and Outcome (PICO) criteria [20], where the population was older adults, the intervention was AI, the comparison was compared or not with other diagnostic methods, and the observation was FS (reporting at least one of the following values: sensitivity, specificity, accuracy, AUC).

The search was conducted using the following keywords and Medical Subject Heading descriptors on PubMed, along with the following search elements on WoS, Scopus, and Google Scholar: “artificial intelligence,” “deep learning,” “machine learning,” “natural language processing,” “neural network,” “unsupervised learning,” “supervised learning,” “frail elderly,” “frailty,” “frail syndrome,” “diagnos*,” “recog,*” “prognosis,” “detect*,” “screening.” Moreover, the Boolean descriptors “AND” and “OR” were also included. The full search strategy is shown in Multimedia Appendix 1.

### Eligibility Criteria

The papers included in this review contained information on the identification and diagnosis of FS in older adults through any type of AI. Textbox 1 shows the specific criteria used for paper inclusion or exclusion.

**Criteria for study selection.**

Inclusion criteria

- Diagnosis, identification, assessment, or classification of frailty syndrome in older adults
- Any type of artificial intelligence
- Papers published in scientific journals
- Individuals older than 65 years
- Study published in any country
- Study published in any year
- English
- Reporting at least on data related to the sensitivity, specificity, accuracy, and area under the curve

Exclusion criteria

- Systematic or scoping reviews
- Studies without human subjects

### Selection of Studies

Four steps were implemented during the study selection process. First, entries were identified on the databases and independently reviewed after searching for and identifying duplicate works. Second, headings and abstracts of all registers were examined to identify those to be eventually included. Third, the full text of all candidate papers was read for determination of their final inclusion or exclusion in this study. Fourth, after selecting the final entries, an in-depth review of the studies in full was conducted. All four steps were independently reviewed by two researchers (AGM and DVD), while any inconsistency detected was discussed and settled by mutual agreement or engaging a third researcher (VPC).

### Data Extraction

According to the PRISMA-ScR guidelines, once the papers had been selected upon assessing that the inclusion criteria had been met, the abstraction of data of interest began, including: (1) general characteristics of the studies, (2) specific classification of different AI models used in each study, (3) characteristics of the data used in each study, and (4) type of data set in each study. The type of data used in each study was specifically indicated, including (5) data related to sensitivity, specificity, accuracy, and AUC; and (6) performance of each model provided by the different papers.

## Results

### Selection of Sources of Evidence

After conducting the search using the keywords and Boolean descriptors mentioned above, a total of 926 papers were identified from different databases. Once duplicate papers were eliminated, a first screening was implemented by analyzing the title and abstract. All papers not meeting the inclusion criteria were excluded; the main cause was the lack of identification or diagnosis of FS in older adults or not using AI. The full text was identified for 37 papers meeting the inclusion criteria.

**Figure 1:**
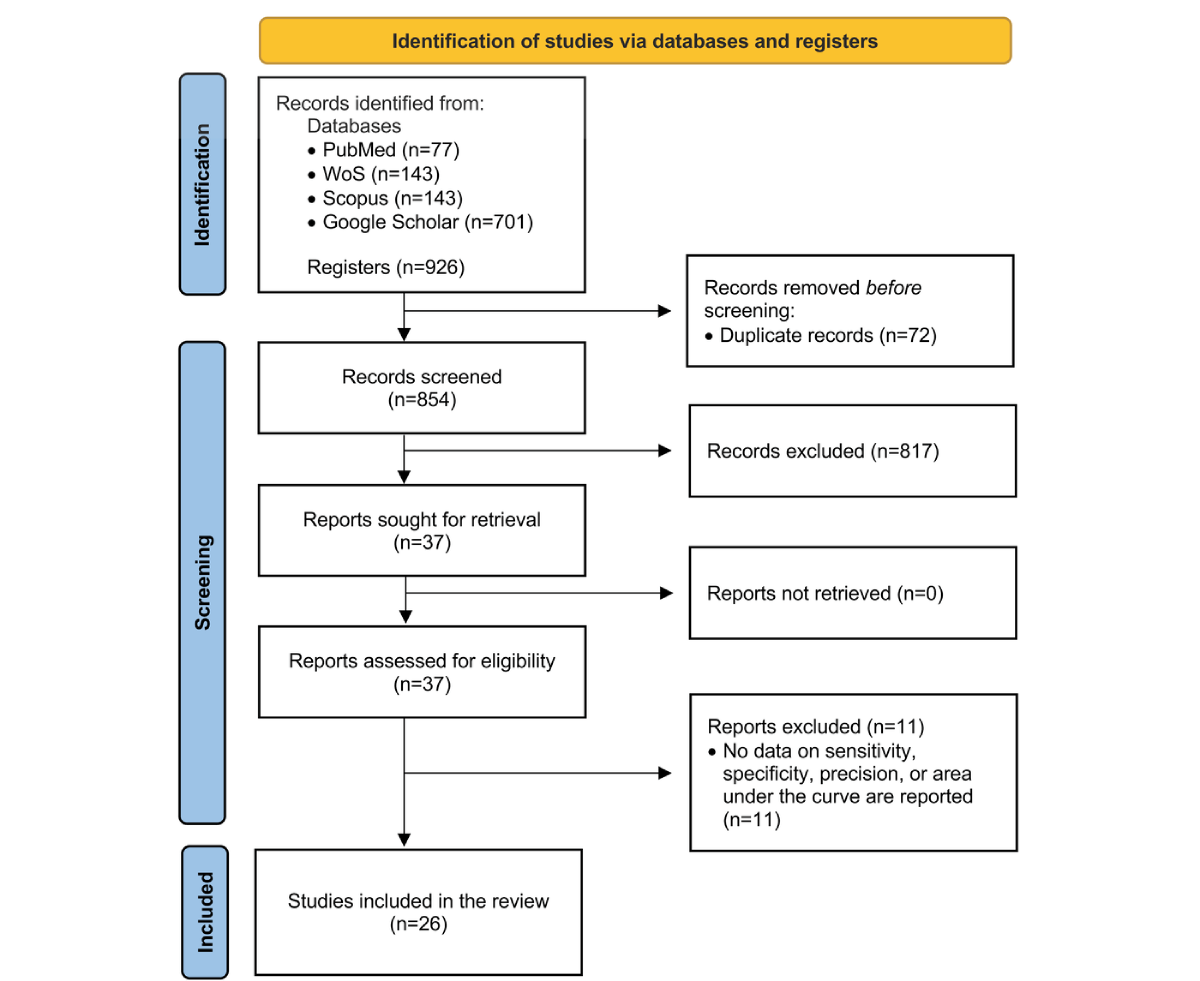
Flow chart of paper selection. WoS: Web of Science.

Of these 37 papers, 11 did not report at least one of sensitivity, specificity, accuracy, or AUC, and were consequently excluded from this review. Finally, 26 articles [21-46] were included in the present review (Figure 1).

### Characteristics of the Included Studies

Among the most relevant countries of publication were the United States, where 30.7% of the studies were published, followed by Korea and Taiwan, with 11.5% each. The years in which more publications were released were 2021 and 2020, accounting for 38.5% and 26.9% of all articles, respectively. With the exception of two articles, the remaining articles identified FS in older adults from a general perspective, without specifying the type of frailty. Machine learning was the type of AI most frequently used for studies amounting for 69.2% of all studies (see Table 1).

**Table 1:** General characteristics of the published research papers on the use of artificial intelligence in the identification and diagnosis of frailty syndrome (N=26).

Characteristics			Studies, n (%)
**Country of publication**			
	United States	8 (30.7)	
	Korea	3 (11.5)	
	Taiwan	3 (11.5)	
	France	1 (3.8)	
	Italy	1 (3.8)	
	Australia	2 (7.6)	
	Spain	2 (7.6)	
	Austria	2 (7.6)	
	Switzerland	1 (3.8)	
	Singapore	1 (3.8)	
	Belgium	1 (3.8)	
	Germany	1 (3.8)	
**Year of publication**			
	2022	4 (15.3)	
	2021	10 (38.5)	
	2020	7 (26.9)	
	2019	2 (7.6)	
	2018	2 (7.6)	
	2013	1 (3.8)	
**Type of frailty**			
	Frailty in older adults	24 (92.3)	
	Social frailty	1 (3.8)	
	Physical frailty	1 (3.8)	
**Type of artificial intelligence**			
	Neural network	3 (11.5)	
	Machine learning	18 (69.2)	
	Deep learning	3 (11.5)	
	Short-term memory network	2 (7.6)	

### AI Algorithms Used

Table 2 shows the detailed classification of the AI algorithms used in the included studies. The information contained was structured by classification models, linear regressions, and deep learning–based models.

**Table 2:** Artificial intelligence models and algorithms, methods, and tools used in the included studies (N=26).

Types of models		Studies, n (%)	References
**Classification models**			
	Support vector machines	8 (30.7)	[21-28]
	Artificial neural network	4 (15.4)	[28-31]
	Multilayer perceptron	2 (7.7)	[22,23]
	Random forest	9 (34.6)	[23,25-28,32-35]
	Linear discriminant analysis	2 (7.7)	[26,36]
	Nearest-neighbor classification	6 (23.1)	[21,22,24,25,35,37]
	Naive Bayes algorithm	6 (23.1)	[21,23,25,26,36,37]
	Extreme gradient boosting	2 (7.7)	[21,32]
	Classification tree algorithm	3 (11.5)	[21,26,32]
	C5.0 algorithm	2 (7.7)	[26,32]
**Regression models**			
	Baseline logistic regression	6 (23.1)	[23,27,28,36,38,39]
	Elastic net method	2 (23.1)	[21,34]
	Regression tree	3 (11.5)	[21,26,32]
	Decision tree	3 (11.5)	[24,28,37]
**Deep learning–based models**			
	Deep neural network	4 (15.4)	[40-43]
	Feedforward neural network	1 (3.8)	[21]
	Shallow neural network	2 (7.7)	[40,44]
	Single-task neural network	1 (3.8)	[34]
	Multitask neural network	1 (3.8)	[34]
	Long short-term memory network	2 (23.1)	[45,46]

### Characteristics of the Data in the Included Studies

Table 3 shows the characteristics of the different data used in the included studies. The information contained was structured by the size of the data set used, type of data (clinical or nonclinical), abstraction source (public/private), and type of subject within the study sample.

**Table 3:** Characteristics of the data included in each study on the diagnosis of frailty syndrome (N=26).

Characteristic		Studies, n (%)
**Size of data set^a^**		
	<100	2 (7.6)
	100-200	3 (11.5)
	200-600	3 (11.5)
	700-1000	1 (3.8)
	1000-2000	1 (3.8)
	>2000	6 (23)
**Type of data**		
	Clinical data	9 (34.6)
	Nonclinical data	17 (65.4)
**Data source**		
	Private	18 (69.2)
	Public	8 (30.8)

^a^11 studies did not state the size of the data set.

### Types of Data Used in the Included Studies

Table 4 shows the characteristics of the different types of data used in the studies included in this review. The information contained was structured by the set of clinical and nonclinical data of subjects under study, with a set of subtypes explored and identified.

**Table 4:** Types of data sets in the included studies (N=26).

Type of data			Studies, n		References
**Clinical data (n=9)**					
	Heart rate dynamics	1		[46]	
	Electronic medical history	6		[24,26-28,32,34]	
	LSNS-6^a^ questionnaire	1		[32]	
	GDS^b^ questionnaire	1		[26]	
	ADL^c^ questionnaire	1		[26]	
	eFS^d^ questionnaire	1		[27]	
	MoCA^e^ test	1		[45]	
	UPSA-B^f^ questionnaire	1		[37]	
**Nonclinical data (n=17)**					
	Activity monitoring through a kinetic sensor	4		[22,35,36,38]	
	Activity monitoring through an inertial sensor in a clinical context	5		[30,31,42,43,45]	
	Activity monitoring through an inertial sensor in a nonclinical context	5		[23,25,29,39,40]	
	Grip strength monitoring	1		[44]	
	Activity monitoring through an ultrasound sensor	1		[33]	
	Activity monitoring through a radar sensor	1		[41]	

^a^LSNS-6: Lubben Social Network Scale.

^b^GDS: Geriatric Depression Scale.

^c^ADL: activities of daily living.

^d^eFS: Edmonton frailty scale.

^e^MoCA: Montreal Cognitive Assessment.

^f^UPSA-B: University of California Davis Performance-based Skills Assessment.

### Statistical Validity of AI Models

Table 5 shows the ratio of different data explored and abstracted in the studies related to accuracy, sensitivity, specificity, and AUC.

**Table 5:** Statistical validity of the tools used (N=26).

Statistics		Studies, n (%)	References
**Accuracy (%)^a^**			
	71-80	7 (26.9)	[26,28,30,35,37-39]
	81-90	7 (26.9)	[21,23,31,36,43,44,46]
	>90	7 (26.9)	[22,24,25,32,40,42,45]
**Sensitivity (%)^b^**			
	<60	1 (3.8)	[27]
	61-74	2 (7.6)	[39,44]
	75-89	7 (26.9)	[21,28,29,34,38,41,46]
	>90	5 (19.2)	[23-26,32]
**Specificity (%)^c^**			
	<70	1 (3.8)	[45]
	71-80	6 (23)	[21,26-28,39,46]
	81-89	3 (11.5)	[24,29,45]
	>90	3 (11.5)	[25,32,44]
**Area under the curve (%)^d^**			
	71-80	4 (15.4)	[26,27,34,39]
	81-89	6 (23.1)	[21,23,29,31,38,46]
	>90	4 (15.4)	[32,33,35,36]

^a^Accuracy was not reported in 5 papers: [27,29,33,34,41].

^b^Sensitivity was not reported in 11 papers: [22,30,31,33,35-37,40,42,43,45].

^c^Specificity was not reported in 13 papers: [22,30,31,33-37,40-43,45].

^d^Area under the curve was not reported in 12 papers: [22,24,25,28,30,37,40-45].

### Performance of Each Model

Table 6 summarizes the AI models and type of data used in each study, along with specific data for sensitivity, specificity, accuracy, and AUC.

**Table 6:** Performance for each artificial intelligence model according to the type of data (N=26).

Type of data and proposed model			Accuracy (%)		Sensitivity (%)		Specificity (%)		AUC^a^ (%)
Heart rate dynamics: long short-term memory [46]			82		83		80		87
**Electronic medical histories**									
	XGBoost^b^ [21]	83.18		78.14		74.41		84.87	
	Support vector machine [24]	93.48		97.83		89.13		NR^c^	
	Elastic net method [34]	NR		80		NR		72	
	Support vector machine [28]	79		77		80		NR	
Electronic medical histories and LSNS-6^d^ questionnaire: C5.0 algorithm [32]			97		97.3		96.7		98.8
Electronic medical histories, and GDS^e^ and ADL^f^ questionnaires: support vector machine [26]			78.47		82.7		71.4		77.1
Electronic medical histories and eFS^g^ questionnaire: logistic regression [27]			NR		54		79		71
MoCA^h^ test and UPSA-B^i^ questionnaire: decision tree [37]			79.2		NR		NR		NR
**Activity monitoring through a kinetic sensor**									
	Baseline logistic regression [38]	73.91		79.37		67.20		82.18	
	Support vector machine [22]	97.5		NR		NR		NR	
	Logistic regression [36]	81		NR		NR		98	
	Nearest-neighbor classification algorithm [35]	71.9		NR		NR		91.9	
**Activity monitoring through an inertial sensor in a clinical context**									
	Long short-term memory network [45]	96.2		NR		NR		NR	
	Artificial neural network [30]	73.3		NR		NR		NR	
	Artificial neural network [31]	90		NR		NR		88	
	Deep neural network [42]	94.63		NR		NR		NR	
	Deep neural network [43]	85.1		NR		NR		NR	
**Activity monitoring through an inertial sensor in a nonclinical context**									
	Deep neural network and shallow neural network [40]	99.72		NR		NR		NR	
	Naive Bayes algorithm [23]	88		91		82		87	
	Logistic regression [39]	73.2		71.8		74.2		79.5	
	Artificial neural network [29]	NR		79.71		86.25		83.22	
	Nearest-neighbor classification algorithm [25]	99.17		97.64		99.47		NR	
Grip strength monitoring: shallow neural network [44]			85.5		67.4		94.2		NR
Activity monitoring through an ultrasound sensor: random forest [33]			NR		NR		NR		96.9
Activity monitoring through a radar sensor: deep neural network [41]			NR		81.02		NR		NR

^a^AUC: area under the curve.

^b^XGBoost: extreme gradient boosting.

^c^NR: not reported.

^d^LSNS-6: Lubben Social Network Scale.

^e^GDS: Geriatric Depression Scale.

^f^ADL: activities of daily living.

^g^eFS: Edmonton Frailty Scale.

^h^MoCA: Montreal Cognitive Assessment.

^i^UPSA-B: University of California San Diego Performance-based Skills Assessment.

## Discussion

### Principal Findings

This scoping review primarily aimed to analyze existing scientific evidence on the use of AI in the identification and diagnosis of FS in older adults, as well as to identify which model provides enhanced accuracy, sensitivity, specificity, and AUC. The outcomes achieved showed that AI might be an acceptable, accurate, and reliable tool for the identification and diagnosis of FS in older adults. However, existing evidence showed highly heterogeneous results as far as the application methods are concerned as well as in relation to the data that need to be included in the programming being implemented.

### AI Models Used in the Diagnosis and Identification of FS

A considerable number of different AI algorithms were identified. Therefore, it would be interesting to find out which types of algorithms have been used and determine which AI features provide enhanced accuracy, sensitivity, specificity, and AUC for identifying and diagnosing FS.

Of the 26 papers included in this review, the most frequently used models were usually the classification models, with random forest models accounting for 34.6% and support vector machine accounting for 30.7% of all models, followed by the nearest-neighbor classification algorithm (23%), naive Bayes algorithm (23%), and baseline logistic regression (23%). Following these, artificial models such as neural network and deep neural network algorithms were also identified (15.4%). Finally, other models were used, but featuring a lower ratio.

### Types of Data Used in AI Models

Another relevant variable of interest was related to the details on the data used in different models. In this sense, these details may be classified into the size of the data set used as well as the used source, mainly nonclinical data and clinical data of patients.

The size of the data set was not reported in numerous papers (42.3%). Among the papers reporting this information, the majority (23%) used a data set size above 2000. Following these, 11.5% used between 200 and 600 data, 11.5% had a data set size between 100 and 200, 7.6% had a data set of less than 100, 3.8% were between 700 and 1000, and 3.8% were between 1000 and 2000.

Most of the studies used nonclinical data (65.4%), which were obtained through different sensors monitoring an activity. The most frequently used sensors were portable sensors and inertial sensors (19.2%), followed by a kinetic sensor (15.4%), radar sensor (3.84%), ultrasound sensor (3.84%), and grip strength sensor (3.84%), represented by one study each.

To a lesser extent, clinical data were also used (34.6%). Most of these studies are based on data abstracted from electronic medical histories (23.1%). Data were also abstracted from questionnaires and heart rate dynamics, although at a lower frequency.

### Accuracy, Sensitivity, Specificity, and AUC of AI Models

The retrieved and examined studies contained at least one of the four main measurements for the performance of a clinical diagnosis of FS based on AI models, including accuracy, sensitivity, specificity, and AUC. The analysis was focused on the indicators identified for diagnostic validity and was based on the type of data, along with the computation model used. The most frequently reported data attaining the highest values in the diagnostic validity of FS were those focused on assessing physical functions by parameterization of kinetics and kinematics.

Using inertial sensors for the monitoring of kinematic variables in clinical contexts was reported in five of the studies [30,31,42,43,45]. Although inertial sensors for kinematic analysis in clinical contexts were among the most frequently used tools for FS identification, most of these studies [30,42,43,45] only reported the data for accuracy, ranging from the lowest accuracy value of 73.3% from Panhwarr et al [30] with an artificial neural network to the highest value of 96.2% from the study by Jung et al [42] based on the deep neural network computational technique. Only Rahemi et al [31] reported the AUC value (88%).

Moreover, the use of inertial sensors for monitoring activities in a nonclinical context was also frequently used among the included studies [23,25,29,39,40]. In four [23,25,29,39] of the five studies using a kinematic data profile, at least three of the four performance metrics of interest were reported. In this sense, irrespective of the programming used, we found that the lowest accuracy value, reported by Park et al [39], was 73.2% and the highest value, reported by Abbas et al [40], was 99.72%. Concerning the sensitivity value, four of the studies reported these data [23,25,29,39], ranging from the highest value of 97.64% in Garcia-Moreno et al [25] to the lowest value of 71.8% in Park et al [39]. Four of the studies in this subgroup reported specificity [23,25,29,39], which ranged from 74.2%, reported by Park et al [39], to 99.47%, reported by Abbaset et al [40]. Finally, within this group of kinematic data, the AUC values ranged from 79.5% in Park et al [39] to 87% in Minici et al [23].

The use of kinetic sensors for activity monitoring was reported in four studies [22,35,36,39]. However, only one of these studies [39] reported all four metrics of diagnostic capacity, attaining values that were lower than those obtained with models based on other data types. Two studies [39,43] reported AUC data equal to or higher than 91.9%, although they did not report information on sensitivity or specificity. Due to the lack of reported information, we cannot confirm data based on kinetics as the most suitable for FS diagnosis. Future research reporting data on sensitivity along with specificity for this type of data profile is needed.

When focusing on the different AI methodologies applied and their diagnostic capacity, heterogeneity was also found in the results. Garcia-Moreno et al [25] showed the best ratios for the diagnostic capacity of FS in older adults by implementing a nearest-neighbor classification algorithm. However, this type of AI was only used in one other study [35] and the findings reported based on kinetic data were not aligned; thus, the identification and diagnosis capacity of this type of AI was lower. In addition, within the AI computation group showing the best results in relation to classification and diagnosis, we found one study [32] that implemented the C5.0 algorithm AI model, which was used for the analysis of patients’ clinical information sourced from their medical records and self-reported details provided by questionnaires. Other AI models that are frequently implemented [26,30,35,41] were support vector machine–based models, showing homogeneous findings with high diagnostic accuracy ratios. Another frequently used model was a neural network [22,23,31,34,44,45], in which heterogeneity was apparent in relation to the statistics used. The diagnostic accuracy, which was the most frequently reported metric, reached above 73.3%. Finally, logistic regression was reported as another AI method for this purpose, which was used in four studies [28,32,42,43], but showed the lowest overall ratios for diagnostic accuracy among all of the AI models reported in the included studies.

### Conventional Models Versus AI for FS Identification

Conventional FS assessment has traditionally been based on two principal sources: (1) self-reported questionnaires such as the Groningen Frailty Indicator, Tilburg Frailty Indicator, Sherbrooke Postal Questionnaire, Vulnerable Elders Survey, and Strawbridge Frailty Questionnaire and (2) clinical assessments measuring frailty, such as the Clinical Frailty Scale, Clinical Global Impression of Change in Physical Frailty, and Short Physical Performance Battery. The latter assessments have been the most frequently used in clinical contexts. More recently, specific FS assessment tools have been developed, such as the Edmonton Frailty Scale, Frailty Index derived from the Comprehensive Geriatric Assessment, and Triage Risk Screening Tool, based on models including different domains [47]. This conventional stream for FS diagnosis explains the fact that numerous models identified in this review used data associated with physical function through movement and other sensors. The combined models are booming tools that imply a longer assessment but a better diagnostic success rate.

These conventional tools showed an average accuracy cut-off point at 83% with respect to FS identification. However, the sensitivity ranged from 56% to 89.5% and the specificity varied between 52% and 91.3% [47]. The results of these conventional models were lower than those achieved by AI for most of the records, with many of the AI computational programming tools obtaining performance values higher than 95% for the accuracy, sensitivity, and specificity metrics. Traditionally, FS diagnosis has mostly been focused on biological variables. In this sense, the identification and classification of geriatric syndromes are extremely likely to fail owing to high variability in the underlying biological factors. Given the multifactorial nature of FS in particular and geriatric syndromes in general, the use of AI and data science is being considered in the future when identifying and diagnosing complex health conditions.

Integrating AI into the identification and diagnosis of FS requires the development and validation of precise algorithms that incorporate multiple data sources such as wearable devices and sensors. To ensure the highest level of integrity, it is crucial to address data quality and privacy concerns. AI should be used as a complementary tool to support clinical judgment rather than as a replacement. After validation, AI systems should be user-friendly and integrated into clinical practice. The performance of AI algorithms should be regularly monitored to ensure their continued relevance and accuracy in clinical practice.

### Strengths and Limitations

To the best of our knowledge, this is the first scoping review analyzing diverse tools for FS identification and diagnosis using AI, as well as the accuracy, sensitivity, specificity, and AUC values from different models.

A set of limitations were identified. First, there is a scarcity of papers reporting all statistical data on accuracy, sensitivity, specificity, and AUC of AI tools. Second, the high heterogeneity shown by the various AI models did not allow us to standardize findings on which type of AI would be the most suitable for having our goal in mind. Another inherent limitation of these AI systems is that their programming, together with the collection of the data to be used, requires a huge amount of expertise and time compared to many of the conventional systems. Finally, a meta-analysis would be interesting to obtain more quantitative information from this scoping review.

### Recommendations for the Future

Future research will be necessary to report all the parameters needed to conveniently establish the validity, accuracy, and reliability of these AI computational systems. Further research is needed, using sufficient data, as well as an analysis based on data cross-checking from different variables, and a meta-analysis could be interesting to offer more qualitative information about the main topic of the present scoping review. A point of interest for future research would be the use of combined diverse data sources rather than only a single type of data, as demonstrated by the diverse studies included in this review. The operational nature of AI implies that the higher the volume of data used and the higher the variety of variables included, the more likely it will be to determine a convenient functioning and learning development when diagnosing multifactorial constructs such as FS. It should be mentioned that all studies included are framed in the development of basic research. It would be interesting for future studies to implement these AI systems through translational research in health systems and different environments.

### Conclusions

This scoping review showed that AI may be an acceptable, accurate, and reliable tool for the identification and diagnosis of FS in older adults. AI could be a useful tool for identifying and diagnosing FS in older adults in both clinical and nonclinical contexts. However, reported evidence shows highly heterogeneous results as far as the application methods are concerned as well as in relation to the data that need to be included in the programming being implemented.

Future research is needed to assess the validity, accuracy, and reliability of AI computational systems using adequate data sources and diverse variables. Combining diverse data sources would be beneficial, and implementation of these systems in health systems and different environments through translational research would be interesting.

The data collected proved that the most well-performing analysis lies in the combined use of electronic medical histories along with kinematic information from inertial sensors monitoring activities in a nonclinical context (activities of daily living). Currently, AI-based computational systems are valid, accurate, and reliable tools that when implemented in health care systems can help to reduce direct and indirect social and health costs associated with dependency and disability.

## Appendix

Multimedia Appendix 1 Detailed search strategies in different databases.

Multimedia Appendix 2 PRISMA checklist.

## Abbreviations

AI: artificial intelligence
AUC: area under the curve
FS: frailty syndrome
PICO: Population/Problem, Intervention, Comparison, and Outcome
PRISMA-ScR: Preferred Reporting Items for Systematic Reviews and Meta-Analysis extension for Scoping Reviews
WoS: Web of Science

## References

[1] World Population Prospects 2019: Highlights (ST/ESA/SER.A/423). United Nations Department of Economic and Social Affairs.

[2] Pison G, Couppié E, Caporali A (2022). The population of the world, 2022. Pop Soc.

[3] Walston J, Hadley E, Ferrucci L, Guralnik J, Newman A, Studenski S, Ershler W, Harris T, Fried LP (2006). Research agenda for frailty in older adults: toward a better understanding of physiology and etiology: summary from the American Geriatrics Society/National Institute on Aging Research Conference on Frailty in Older Adults. J Am Geriatr Soc.

[4] Rockwood K, Mitnitski A (2007). Frailty in relation to the accumulation of deficits. J Gerontol A Biol Sci Med Sci.

[5] (2015). Informe mundial sobre el envejecimiento y la salud: resumen. World Health Organization.

[6] Crocker TF, Brown L, Clegg A, Farley K, Franklin M, Simpkins S, Young J (2019). Quality of life is substantially worse for community-dwelling older people living with frailty: systematic review and meta-analysis. Qual Life Res.

[7] Shega J, Dale W, Andrew M, Paice J, Rockwood K, Weiner DK (2012). Persistent pain and frailty: a case for homeostenosis. J Am Geriatr Soc.

[8] Fried L, Tangen C, Walston J, Newman AB, Hirsch C, Gottdiener J, Seeman T, Tracy R, Kop WJ, Burke G, McBurnie MA, Cardiovascular Health Study Collaborative Research Group (2001). Frailty in older adults: evidence for a phenotype. J Gerontol A Biol Sci Med Sci.

[9] Morley JE, Haren MT, Rolland Y, Kim MJ (2006). Frailty. Med Clin North Am.

[10] Parvaneh S, Mohler J, Toosizadeh N, Grewal G, Najafi B (2017). Postural transitions during activities of daily living could identify frailty status: application of wearable technology to identify frailty during unsupervised condition. Gerontology.

[11] Caballero-García JC, Bénitez Rivero J (2012). Manual de atención al anciano desnutrido en el nivel primario de salud 2011 (Manual of care for the malnourished elderly at the primary health level 2011). EnfermiaAPS.

[12] Weiss A, Herman T, Plotnik M, Brozgol M, Giladi N, Hausdorff JM (2011). An instrumented timed up and go: the added value of an accelerometer for identifying fall risk in idiopathic fallers. Physiol Meas.

[13] Galán-Mercant A, Cuesta-Vargas AI (2013). Differences in trunk accelerometry between frail and nonfrail elderly persons in sit-to-stand and stand-to-sit transitions based on a mobile inertial sensor. JMIR Mhealth Uhealth.

[14] Galán-Mercant A, Cuesta-Vargas AI (2015). Clinical frailty syndrome assessment using inertial sensors embedded in smartphones. Physiol Meas.

[15] Bandeen-Roche K, Xue Q, Ferrucci L, Walston J, Guralnik J, Chaves P, Zeger S, Fried LP (2006). Phenotype of frailty: characterization in the women's health and aging studies. J Gerontol A Biol Sci Med Sci.

[16] Chumha N, Funsueb S, Kittiwachana S, Rattanapattanakul P, Lerttrakarnnon P (2020). An artificial neural network model for assessing frailty-associated factors in the Thai population. Int J Environ Res Public Health.

[17] Johnson KW, Torres Soto J, Glicksberg BS, Shameer K, Miotto R, Ali M, Ashley E, Dudley JT (2018). Artificial intelligence in cardiology. J Am Coll Cardiol.

[18] Goodfellow I, Bengio Y, Courville A (2016). Deep Learning (Adaptive Computation and Machine Learning series).

[19] Tricco A, Lillie E, Zarin W, O'Brien KK, Colquhoun H, Levac D, Moher D, Peters MDJ, Horsley T, Weeks L, Hempel S, Akl EA, Chang C, McGowan J, Stewart L, Hartling L, Aldcroft A, Wilson MG, Garritty C, Lewin S, Godfrey CM, Macdonald MT, Langlois EV, Soares-Weiser K, Moriarty J, Clifford T, Tunçalp Ö, Straus SE (2018). PRISMA Extension for Scoping Reviews (PRISMA-ScR): checklist and explanation. Ann Intern Med.

[20] Schardt C, Adams M, Owens T, Keitz S, Fontelo P (2007). Utilization of the PICO framework to improve searching PubMed for clinical questions. BMC Med Inform Decis Mak.

[21] Aponte-Hao S, Wong S, Thandi M, Ronksley P, McBrien K, Lee J, Grandy M, Mangin D, Katz A, Singer A, Manca D, Williamson T (2021). Machine learning for identification of frailty in Canadian primary care practices. Int J Popul Data Sci.

[22] Akbari G, Nikkhoo M, Wang L, Chen C, Han D, Lin Y, Chen HB, Cheng CH (2021). Frailty level classification of the community elderly using Microsoft Kinect-based skeleton pose: a machine learning approach. Sensors (Basel).

[23] Minici D, Cola G, Giordano A, Antoci S, Girardi E, Bari MD, Avvenuti M (2022). Towards automated assessment of frailty status using a wrist-worn device. IEEE J Biomed Health Inform.

[24] Ambagtsheer R, Shafiabady N, Dent E, Seiboth C, Beilby J (2020). The application of artificial intelligence (AI) techniques to identify frailty within a residential aged care administrative data set. Int J Med Inform.

[25] Garcia-Moreno FM, Bermudez-Edo M, Garrido JL, Rodríguez-García E, Pérez-Mármol JM, Rodríguez-Fórtiz MJ (2020). A microservices e-Health system for ecological frailty assessment using wearables. Sensors (Basel).

[26] Hassler A, Menasalvas E, García-García FJ, Rodríguez-Mañas L, Holzinger A (2019). Importance of medical data preprocessing in predictive modeling and risk factor discovery for the frailty syndrome. BMC Med Inform Decis Mak.

[27] Le Pogam MA, Seematter-Bagnoud L, Niemi T, Assouline D, Gross N, Trächsel B, Rousson V, Peytremann-Bridevaux I, Burnand B, Santos-Eggimann B (2022). Development and validation of a knowledge-based score to predict Fried's frailty phenotype across multiple settings using one-year hospital discharge data: The electronic frailty score. EClinicalMedicine.

[28] Tarekegn A, Ricceri F, Costa G, Ferracin E, Giacobini M (2020). Predictive modeling for frailty conditions in elderly people: machine learning approaches. JMIR Med Inform.

[29] Chang Y, Lin C, Lin P, Chen C, Lee R, Huang J, Tsai T (2013). eFurniture for home-based frailty detection using artificial neural networks and wireless sensors. Med Eng Phys.

[30] Panhwarr Y, Naghdy F, Stirling D, Naghdy G, Potter J (2020). Quantitative frailty assessment based on kinematic parameters of daily living activities. Annu Int Conf IEEE Eng Med Biol Soc.

[31] Rahemi H, Nguyen H, Lee H, Najafi B (2018). Toward smart footwear to track frailty phenotypes-using propulsion performance to determine frailty. Sensors (Basel).

[32] Kuo K, Talley P, Kuzuya M, Huang CH (2019). Development of a clinical support system for identifying social frailty. Int J Med Inform.

[33] Ziegl A, Hayn D, Kastner P, Loffler K, Weidinger L, Brix B, Goswami N, Schreier G (2020). Machine learning based walking aid detection in timed up-and-go test recordings of elderly patients. Annu Int Conf IEEE Eng Med Biol Soc.

[34] Martin J, Crane-Droesch A, Lapite FC, Puhl JC, Kmiec TE, Silvestri JA, Ungar LH, Kinosian BP, Himes BE, Hubbard RA, Diamond JM, Ahya V, Sims MW, Halpern SD, Weissman GE (2021). Development and validation of a prediction model for actionable aspects of frailty in the text of clinicians' encounter notes. J Am Med Inform Assoc.

[35] Kraus M, Saller M, Baumbach S, Neuerburg C, Stumpf U, Böcker W, Keppler AM (2022). Prediction of physical frailty in orthogeriatric patients using sensor insole-based gait analysis and machine learning algorithms: cross-sectional study. JMIR Med Inform.

[36] Goonawardene N, Tan HP, Tan LB, Zhou J, Salvendy G (2018). Unobtrusive detection of frailty in older adults. Human Aspects of IT for the Aged Population. Applications in Health, Assistance, and Entertainment. ITAP 2018. Lecture Notes in Computer Science, vol 10927.

[37] Kumar S, Du C, Graham S, Nguyen T (2021). Using machine learning to predict frailty from cognitive assessments. Annu Int Conf IEEE Eng Med Biol Soc.

[38] Park C, Mishra R, Sharafkhaneh A, Bryant M, Nguyen C, Torres I, Naik A, Najafi B (2021). Digital biomarker representing frailty phenotypes: the use of machine learning and sensor-based sit-to-stand test. Sensors (Basel).

[39] Park C, Mishra R, Golledge J, Najafi B (2021). Digital biomarkers of physical frailty and frailty phenotypes using sensor-based physical activity and machine learning. Sensors (Basel).

[40] Abbas M, Somme D, Le Bouquin Jeannes R (2020). Machine learning-based physical activity tracking with a view to frailty analysis.

[41] Zhang Y, Babarinde O, Han P, Wang X, Karsmakers P, Schreurs D, Verschueren S, Vanrumste B (2021). Automatically segmenting physical performance test items for older adults using a Doppler radar: a proof of concept study. IEEE Access.

[42] Jung D, Nguyen M, Park M, Kim M, Won C, Kim J, Mun KR (2020). Walking-in-place characteristics-based geriatric assessment using deep convolutional neural networks. Annu Int Conf IEEE Eng Med Biol Soc.

[43] Arshad M, Jung D, Park M, Shin H, Kim J, Mun KR (2021). Gait-based frailty assessment using image representation of IMU signals and deep CNN.

[44] Pérez E, Rangel J, Musté M, Pérez C, Macho O, del Corral Guijarro FS, Somoano A, Gianella C, Ramírez L, Català A, Rojas I, Joya G, Català A (2021). Frailty level prediction in older age using hand grip strength functions over time. Advances in Computational Intelligence. IWANN 2021. Lecture Notes in Computer Science, vol 12862.

[45] Jung D, Kim J, Kim M, Won CW, Mun K (2021). Frailty assessment using temporal gait characteristics and a long short-term memory network. IEEE J Biomed Health Inform.

[46] Eskandari M, Parvaneh S, Ehsani H, Fain M, Toosizadeh N (2022). Frailty identification using heart rate dynamics: a deep learning approach. IEEE J Biomed Health Inform.

[47] Huang EY, Lam SC (2021). Review of frailty measurement of older people: evaluation of the conceptualization, included domains, psychometric properties, and applicability. Aging Med (Milton).

