# Supplementary material for: Use of Artificial Intelligence in the Identification and Diagnosis of Frailty Syndrome in Older Adults: Scoping Review
Source: J Med Internet Res. 2023 Oct 20;25:e47346. doi: 10.2196/47346 (PMC10625070; doi:10.2196/47346)
Supplement: Multimedia Appendix 1 [file jmir_v25i1e47346_app1.pdf]

Appendix 1. Detailed Search Strategies in different Databases	
**Databases**	**Search Strategies**
**Pubmed**	("Artificial Intelligence*" OR "Deep Learning" OR "Machine Learning" OR "Natural Language Processing" OR neural network* OR "unsupervised learning" OR "supervised learning") AND ("Frail Elderly" [MeSH] OR Frailty OR "Frail syndrome") AND (diagnos* OR recog* OR prognosis OR detect* OR screening*)
**Google Schoolar**	"Artificial Intelligence*" OR "Deep Learning" OR "Machine Learning" OR "Natural Language Processing" OR "neural network" OR "unsupervised learning" OR "supervised learning" AND "Frail Elderly" OR Frailty OR "Frail syndrome" AND diagnos OR recog OR prognosi
**Scopus**	("Artificial Intelligence*" OR "Deep Learning" OR "Machine Learning" OR "Natural Language Processing" OR neural network* OR "unsupervised learning" OR "supervised learning") AND ("Frail Elderly" [MeSH] OR Frailty OR "Frail syndrome") AND (diagnos* OR recog* OR prognosis OR detect* OR screening*)
**Web Of Science**	("Artificial Intelligence*" OR "Deep Learning" OR "Machine Learning" OR "Natural Language Processing" OR neural network* OR "unsupervised learning" OR "supervised learning") AND ("Frail Elderly" [MeSH] OR Frailty OR "Frail syndrome") AND (diagnos* OR recog* OR prognosis OR detect* OR screening*)
